# An Interdisciplinary Weight Loss Program Improves Body Composition and Metabolic Profile in Adolescents With Obesity: Associations With the Dietary Inflammatory Index

**DOI:** 10.3389/fnut.2019.00077

**Published:** 2019-06-03

**Authors:** Yasmin Alaby Martins Ferreira, Ana Claudia Pelissari Kravchychyn, Sofia de Castro Ferreira Vicente, Raquel Munhoz da Silveira Campos, Lian Tock, Lila Missae Oyama, Valter Tadeu Boldarine, Deborah Cristina Landi Masquio, David Thivel, Nitin Shivappa, James R. Hébert, Ana R. Dâmaso

**Affiliations:** ^1^Post Graduate Program of Nutrition, Escola Paulista de Medicina, Universidade Federal de São Paulo, São Paulo, Brazil; ^2^Therapeutic Resources Laboratory, Department of Physiotherapy, Universidade Federal de São Carlos (UFSCar), São Carlos, Brazil; ^3^Post Graduate Program of Nutrition, Universidade Federal de São Paulo, São Paulo, Brazil; ^4^Department of Physiology, Escola Paulista de Medicina, Universidade Federal de São Paulo, São Paulo, Brazil; ^5^Centro Universitário São Camilo, São Paulo, Brazil; ^6^Clermont Auvergne University, EA 3533, Laboratory of the Metabolic Adaptations to Exercise Under Physiological and Pathological Conditions (AME2P), Clermont-Ferrand, France; ^7^CRNH-Auvergne, Clermont-Ferrand, France; ^8^Cancer Prevention and Control Program, University of South Carolina, Columbia, SC, United States; ^9^Department of Epidemiology and Biostatistics, Arnold School of Public Health, University of South Carolina, Columbia, SC, United States; ^10^Connecting Health Innovations LLC (CHI), Columbia, SC, United States

**Keywords:** inflammatory diet, cardiometabolic risk, interdisciplinary therapy, obesity, inflammation

## Abstract

**Background and Aims:** The prevalence of overweight and obesity consitutes a global epidemic and it is growing around the world. Food and nutrition are essential requirements for promoting health and protecting against non-communicable chronic diseases, such as obesity and cardiovascular disease. Specific dietary components may modulate inflammation and oxidative stress in obese individuals. The Dietary Inflammatory Index (DII®) was developed to characterize the anti- and pro-inflammatory effects of individuals' diet. Few studies have investigated the role of diet-associated inflammation in adolescents with obesity. The present study aims to investigate the effects of an interdisciplinary weight loss therapy on DII scores and cardiometabolic risk in obese adolescents and possibles correlations.

**Methods:** A total of 45 volunteers (14–19 years old) were recruited and enrolled for long-term interdisciplinary therapy including clinical, nutritional, psychological counseling, and exercise training. Adolescents had access to videos about health education weekly. Body composition and inflammatory and serum profiles were evaluated at baseline and after intervention. The food intake was obtained by 24-h food recall. Data was used to calculate energy-adjusted DII (E-DII) scores. Negative scores indicate an anti-inflammatory diet and positive scores indicates a pro-inflammatory diet. The sample was divided according to whether individuals increased or decreased E-DII scores after therapy.

**Results:** After therapy the body mass index (BMI), body weight, body fat, abdominal, waist, neck, and hip circumferences decreased significantly. The mean of high-density lipoprotein cholesterol (HDL-c) increased after the therapy. There was found an improvement of inflammatory and cardiometabolic parameters. In exploratory analyses, this occurred mainly when the EDII improved.

**Conclusion:** Long-term interdisciplinary therapy combined with a health education website improved inflammatory serum markers in obese adolescents. Reduction in DII scores was associated with reduction of cardiometabolic parameters, suggesting that an anti-inflammatory diet may be an effective strategy to prevent and treat obesity and related comorbidities.

**Trial:** http://www.ensaiosclinicos.gov.br/rg/RBR-6txv3v/, Register Number: RBR-6txv3v

## Introduction

Obesity is a multifactorial disease that requires interdisciplinary therapy and preventive strategies. Obesity-related comorbidities, such as diabetes and cardiovascular disease, currently represent the main costs incurred by health care systems (1, 2). The prevalence of obesity in children and adolescents is increasing worldwide. In Brazil, 20.5% of adolescents between 10 and 19 years old are overweight and 4.9% are obese (3, 4).

Obesity is not just a matter of aesthetics and body image. It also decreases life expectancy and results in cancer, cardiovascular diseases, hypertension, diabetes, steatosis, characterizing metabolic syndrome; all together raising mortality risks (2, 5–7).

Adipose tissue releases adipokines that have specific functions, among them; adiponectin may attenuate inflammatory response and results in beneficial effects on cardiovascular disorders. However, studies indicate that obese individuals present lower concentrations of adiponectin, and concomitantly they are in a state of hyperleptinemia, favoring inflammation. The change in homeostasis and release of cytokines, characterize obesity as a condition of persistent low-grade inflammation, which is closely associated with obesity-related comorbidities (8–11).

Leptin is one of the main factors involved in the energy balance regulation. Obese patients show leptin-resistance that, in turn, can favor inflammatory state (12). In fact, leptin resistance in obese adolescents could influence an expansion of adipose tissue by increasing the release of leptin to supply the resistance, thus leading to problems with weight loss, and contributing to inflammation (13). Therefore, the adiponectin/leptin ratio is an interesting and pertinent indicator relating to adipose tissue dysfunction (14).

While food intake and eating habits are key factors for the promotion of health and prevention of weight gain and cardiovascular disease, some specific dietary components may modulate the inflammation and oxidative stress in obese individuals. Indeed, several studies have observed associations between inflammatory diets and comorbidities, such as obesity, metabolic disorders, intestinal dysbiosis, Parkinson's disease, cancer, depression, and bone weakness (15–23).

The Dietary Inflammatory Index (DII®) was created in 2009 to quantify the effect of diet on inflammatory potential, which was further improved and validated by Shivappa et al. (24). A literature review in 2010 encompassing 1,943 papers studied the association between each of the six inflammatory markers (Interleukin-1β, Interleukin-4, Interleukin-6, Interleukin-10, Tumor Necrosis Factor-α, and C-reactive protein) and 45 food parameters. Negative scores suggest that the meal or ingested food may have anti-inflammatory effects, while positive values indicate pro-inflammatory effects (24). Specifically, among South American population, DII has been tested with colorectal and prostate cancers in Argentina and multiple sclerosis and metabolic syndrome in Brazil (25–27).

Previously, a pro-inflammatory diet has been validated with inflammatory markers among adolescents and has been shown to be associated with increased risk of childhood obesity, cardiometabolic risk, depression, and stress levels among adolescents (28–32). Accordingly, studies conducted in adolescents have indicated that elevated intake of sugar-sweetened soft drinks, refined grains, processed foods, and low intake of vegetables and fiber may increase the incidence of premenopausal breast cancer and cardiometabolic risks (30, 33, 34).

Interesting, multi and interdisciplinary programs have been conducted in adolescents to promote obesity management and long-term weight loss therapies have been shown to produce favorable changes in metabolic parameters and inflammation. While the effects of exercise training are pretty well described, it remains so far unclear how diet can influence obesity related inflammatory markers, such as, adiponectin and leptin in adolescents (11, 35–39).

This study was designed to investigate the effects of an interdisciplinary therapy combined online with support (i.e., web-based health education) on inflammatory markers and to verify possible correlations between energy-adjusted dietary inflammatory index (E-DII) with inflammatory markers in obese adolescents. We hypothesized that the therapy will improve body composition and inflammatory biomarkers, and that reduction of inflammatory biomarkers is correlated with changes in E-DII.

## Materials and Methods

### Population

Forty-five pubertal obese adolescents (21 boys, 24 girls) were enrolled in this study. The study was announced in the media (journals, magazines, radio, and TV) and the adolescents were selected according to the inclusion and exclusion criteria. The inclusion criteria were a body mass index (BMI) *p* > 95th percentile (40); age between 14 and 19 years and post-pubertal Tanner Stage ≥V (41). Exclusion criteria were cardiovascular disease, musculoskeletal problems that limited physical exercise, autoimmune diseases, genetic, metabolic or endocrine disease and chronic alcohol consumption and tabaco consumption and also who used drugs/medicaments and/or nutritional supplements that alter the metabolism during the last 6 months, and who did not have access to electronic means. Predicting a dropout of ~20%, 57 adolescents started the interdisciplinary therapy and 45 adolescents completed the intervention. The main reasons for the treatment dropout were job opportunities and school, followed by financial and family problems.

**Figure 1:**
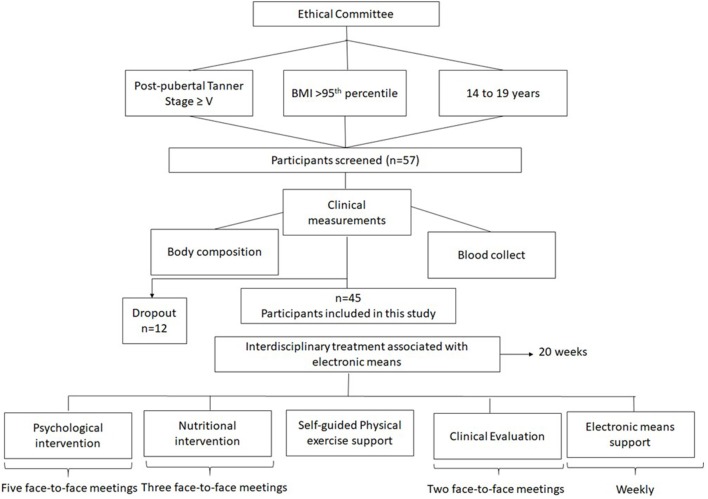
Study design.

At initial interview the protocol was shared with the volunteers, who had received a parental consent form to participate in an interdisciplinary weight loss program. The study was developed in accordance with the Declaration of Helsinki and was approved by the Federal University to Sao Paulo ethics committee (#0448/2018). Brazilian Clinical Trial registration number: RBR-6txv3v. The Figure 1 shows the study design.

### Anthropometric Measurements and Body Composition

Body weight was measured using light clothes and barefoot on a Filizola® scale to 0.1 kg and capacity of 180 kg. Height was measured using to a wall-mounted height board (Sanny®) to the nearest 0.1 cm. Body mass index was then calculated as body weight (kg)/height (meter)^2^. Waist circumference and abdominal circumference were measured with a flexible and inelastic tape. Body composition was measured by a Bio-impedance meter (BIA) and the basal metabolic rate was estimate based on body composition provide by the device BIODYNAMICS 310e (TBW®), that has accuracy: Correlation of *R* = 0.98 with Hydrostatic Weighing and accuracy of ± 1% in Resistance reading.

### Serum Analysis

Blood samples were collected after an overnight 12-h fast. Plasma insulin concentration was measured by radioimmunoassay. According to Schwimmer et al. (42), normal plasma levels of fasting insulin are lower than 17 uU/ml. Plasma glucose was measured by an enzymatic method with a UV-Visible spectrophotometer and interpreted according to the established criteria by the American Diabetes Association (43). Total cholesterol, cholesterol fractions and triglycerides (TG) were analyzed using colorimetric comparative method. The cut-off limits for total cholesterol, LDL-c, HDL-c, and triglycerides were established according to the Brazilian Society of Cardiology (2013) (44). Insulin resistance (HOMA-IR) and insulin sensitivity (QUICKI) were estimated by evaluation model obtained using the formulas proposed by Matthews et al. (45) and Katz et al. (46), respectively:

HOMA-IR: Insulin fasted (Uμ/ml) x fasted blood glucose (mmol/ml)/22.5
QUICKI: 1/(log fasting insulin (Uμ/ml) + log fasting glycaemia (mg/dl)

Adiponectin and Leptin were measured with enzyme-linked immunosorbent assay (ELISA) kit from R&D Systems (Minneapolis, MN, USA).

### Interdisciplinary Therapy Associated With Web-Based Educational Health Approach

Endocrinologists, nutritionists, psychologists, and exercise physiologists developed the interdisciplinary weight loss therapy. In the present investigation, the volunteers completed five face-to-face meetings, including individual and group sessions and during 20-weeks intervention they had access to videos with health topics based on *e-book: Saber Emagrecer* Dâmaso (47). Weekly, body weight was monitored by self-report and photo of their scales. The researchers advised that body weight should be measured on the same equipment to avoid variability among the equipment. Applications related to android mobiles were used to assist in the monitoring of physical exercise and nutritional prescription. Participants also were contacted by phone or email every week in order to motivate them and eventually answer their questions during the intervention period.

### Clinical Therapy

Obese adolescents visited the endocrinologist at the beginning and after therapy to address the health and clinical parameters, including family history.

### Psychological Therapy

Once a month, the psychologist discussed important themes about obesity, including, bulimia, anorexia, self-esteem, and anxiety in groups.

### Exercise-Training

The physical activity level was monitored at the initial evaluation and every 5 weeks by the International Physical Activity Questionnaire (IPAQ)—short version (48). A self-guided method was applied, in which the adolescents chose exercises related to personal pleasure (49). In all face-to-face meetings a professional guided the adolescent to do exercise at least 3 times/week and duration of 1 h/session. Every week mobile apps were used to monitor the physical exercise.

### Nutritional Therapy

Different themes were discussed in personal sessions and online program weekly, including: food labels, diet and light foods, weight loss diets, good food choices on weekends, and celebrations. No supplements were recommended.

Daily energy intake was assessed using self-reported 24-h food recalls (24 HR) completed at baseline and at the end of the 20-week intervention. Hypocaloric diet was prescribed according to age and gender, reducing caloric intake between 300 and 500 kcal/day, respecting the distribution of macronutrients proposed by the Institute of Medicine (50). The DIETSMART^®^ program was used to analyze dietary intake and calculate nutrients intake to calculate the E-DII per 4,184 kJ (1,000 kcal) of food consumed (51). Lower values indicate an anti-inflammatory diet and higher values represent pro-inflammatory diet (24). Twenty -four nutrients were used to calculate the E-DII score (Carbohydrate, Protein, Fat, Fiber, Cholesterol, saturated fat, mono-unsaturated fat, poly-unsaturated fat, Omega3, Omega6, Niacin, Thiamin, Riboflavin Vitamins (A, B12, B6, C, D, E), Iron, Magnesium, Zinc, Selenium, Folic Acid, and Beta-carotene.

### Statistical Analysis

Statistical analysis was performed using the program STATISTICA version 7.0 for Windows. The accepted significant value was α < 5%. Data normality was verified with the Kolmogorov-Smirnov test. Parametric data were expressed as mean ± SD, and non-parametric data was normalized using *Z*-scores. To analyze the effects of intervention and difference between the groups according to the E-DII response: increased the E-DII or decreased E-DII, ANOVA for repeated measures (ANOVA two-way) followed by Fischer *post hoc* test were used.

Delta values (Δ) were used for the statistical analysis obtained from the difference between the after therapy and baseline values for each variable: Δ variable = after therapy value–baseline value. Comparison of the delta values between the groups was performed by *t*-test independent by groups. Product moment; i.e., Pearson correlation coefficients were computed.

Using the G^*^Power® 3.0.10 program, it was obtained a sample size of 46 voluntaries, considering the statistical analysis realized by ANOVA for repeated measures (ANOVA two-way). The effect size 0.30, power 80%, based on E-DII and two groups and two times of evaluation (baseline and after 20 weeks of intervention).

## Results

### Effects of Therapy in Anthropometric Measurements and Body Composition

The study population consisted of 45 adolescents. Assessment of anthropometric measurements and body composition at the beginning and after therapy are presented. The therapy was effective in reducing body weight, BMI, waist circumference, neck circumference, hip circumference and body fat mass (% and kg; Table 1).

**Table 1:** Anthropometric measurements and body composition before and after therapy.

**variables**	**All (*****n*** **=** **45)**		**Increased E-DII (*****n*** **=** **23)**		**Decreased E-DII (*****n*** **=** **22)**		***Effect size***	***Observed power***
	**Baseline**	**After therapy**	**Baseline**	**After therapy**	**Baseline**	**After therapy**		
Body weight (kg)	109.82 ± 15.78	104.29 ± 15.05^a^	112.29 ± 16.94	107.57 ± 16.9^a^	107.24 ± 14.41	100.86 ± 12.16^a^	0.59	0.99
BMI (Kg/m^2^)	38.06 ± 4.66	35.91 ± 4.72^a^	39.24 ± 4.73	37.39 ± 4.72^a^	36.83 ± 4.34	34.37 ± 4.30^a,c^	0.63	0.99
Waist Circumference (cm)	109.81 ± 10.50	105.18 ± 10.86^a^	110.79 ± 10.71	106.77 ± 11.14^a^	108.79 ± 10.43	103.51 ± 10.58^c^^a^	0.41	0.99
Abdominal circumference (cm)	118.19 ± 10.75	110.44 ± 18.00^a^	119.20 ± 9.66	113.98 ± 9.31	117.14 ± 11.92	106.75 ± 23.68^a^	0.22	0.92
Neck circumference (cm)	39.69 ± 3.73	38.3 ± 3.47^a^	39.40 ± 4.03	38.23 ± 4.06^a^	40.00 ± 3.45	38.37 ± 2.81^a^	0.51	0.99
Hip circumference (cm)	124.96 ± 9.29	120.94 ± 8.46^a^	127.46 ± 8.80	123.32 ± 8.28^a^	122.35 ± 9.24	118.45 ± 8.08^a^	0.41	0.99
Body fat mass (%)	*37.66*±5.12	36.14 ± 5.09^a^	38.40 ± 5.27	37.75 ± 4.45	36.88 ± 4.96	34.46 ± 5.27^a,c^	0.23	0.94
Body fat mass (kg)	41.65 ± 8.78	37.70 ± 8.04^a^	43.33 ± 9.36	40.53 ± 8.40^a^	39.90 ± 7.97	34.73 ± 6.58^a,c^	0.45	0.99
Fat free mass (kg)	68.17 ± 10.70	66.63 ± 10.28^a^	68.95 ± 10.86	67.13 ± 11.04^a^	67.35 ± 10.71	66.11 ± 9.66^a^	0.23	0.94
Fat free mass (%)	62.34 ± 5.12	63.85 ± 5.09	61.59 ± 5.27	62.24 ± 4.45	63.12 ± 4.95	65.53 ± 5.27^a,c^	0.23	0.94
BMR (kcal)	2075.58 ± 322.67	2025.56 ± 312.38^a^	2096.43 ± 330.30	2040.65 ± 335.11	2053.77 ± 320.75	2009.77 ± 293.75	0.26	0.96

BMI (Body Mass Index), BMR (Basal Metabolic Rate);

a statistic difference between baseline and after therapy of obese adolescents (p < 0.05);

c *statistic difference between the groups in the end of therapy*.

The subgroup of individuals that decreased E-DII showed significantly greater decrease in BMI, body fat mass (kg and %) and increase in fat free mass (%) after therapy. The BMI, body weight (kg), waist circumference, neck and hip circumference decreased in both groups (Table 1).

### Effects of Therapy on Biochemical Parameters

Considering the biochemical parameters, only HDL-C was shown to significantly increase (*p* < 0.05) while all the other metabolic parameters remained unchanged. On the other hand, the subgroup with decreased E-DII had increase in QUICKI and reduction in glycaemia, VLDL-c, triglycerides, and HOMA-IR. The therapy was effective in increasing the HDL-c even in the group that increased the E-DII.

### Effects of Therapy on Adipokines

The leptin decreased significantly after the intervention, while adiponectin/leptin ratio increased in all adolescents. At baseline, the leptin concentration was statistically different between adolescents who increased and decreased the energy-adjusted dietary inflammatory index (Table 2). Adolescents who decreased the E-DII reduced leptin and increase the adiponectin/leptin ratio after the therapy.

**Table 2:** Biochemical parameters and adipokines of obese adolescents before and after therapy.

**variables**	**All (*****n*** **=** **45)**		**Increased E-DII (*****n*** **=** **23)**		**Decreased E-DII (*****n*** **=** **22)**		***Effect size***	***Observed power***
	**Baseline**	**After therapy**	**Baseline**	**After therapy**	**Baseline**	**After therapy**		
Glycemia (mg/dL)	90.53 ± 7.83	88.73 ± 7.51	91.26 ± 9.21	91.04 ± 6.71	89.77 ± 6.20	86.32 ± 7.67^c^	0.03	0.21
Total Cholesterol (mg/dL)	164.53 ± 34.58	160.98 ± 38.97	160.78 ± 39.93	165.22 ± 43.00	168.45 ± 28.34	156.55 ± 34.70	0.009	0.09
HDL-C (mg/dL)	40.51 ± 8.43	43.80 ± 8.58^a^	41.52 ± 9.37	45.48 ± 9.29^a^	39.45 ± 7.39	42.05 ± 7.58	0.23	0.94
Non-HDL (mg/dL)	124.02 ± 31.66	113.80 ± 36.41	119.39 ± 35.40	113.13 ± 38.84	128.86 ± 27.18	114.50 ± 34.59	0.06	0.40
LDL-c (mg/dL)	99.51 ± 31.36	93.80 ± 32.91	92.74 ± 33.81	92.35 ± 37.76	106.59 ± 27.57	95.32 ± 27.77	0.03	0.21
VLDL-c (mg/dL)	24.44 ± 9.18	22.38 ± 10.92	26.52 ± 10.50	25.43 ± 10.58	22.27 ± 7.17	19.18 ± 10.57^c^	0.03	0.25
TG (mg/dL)	121.96 ± 45.76	111.67 ± 54.33	132.57 ± 52.38	126.65 ± 52.88	110.86 ± 35.50	96.00 ± 52.45^c^	0.03	0.25
Insulin (Uμ/mL)	20.02 ± 12.43	17.83 ± 13.03	21.65 ± 16.16	21.45 ± 16.44	18.31 ± 6.66	14.04 ± 6.59	0.03	0.24
HOMA-IR	4.51 ± 2.84	3.98 ± 3.04	4.90 ± 3.66	4.86 ± 3.80	4.10 ± 1.58	3.06 ± 1.59^c^	0.03	0.2
QUICKI	0.31 ± 0.02	0.32 ± 0.03	0.31 ± 0.03	0.31 ± 0.02	0.31 ± 0.02	0.33 ± 0.03^a^^,^ ^c^	0.08	0.51
Adiponectin (μg/mL)	2.67 ± 2.06	3.64 ± 3.27	2.51 ± 1.53	3.90 ± 3.74	2.83 ± 2.52	3.36 ± 2.75	0.08	0.47
Leptin (ng/mL)	63.74 ± 41.37	49.80 ± 41.38^a^	75.78 ± 47.96	62.49 ± 48.89	51.17 ± 29.20^b^	36.54 ± 26.88^c^	0.23	0.94
Adipo/lep	0.06 ± 0.05	0.13 ± 0.18^a^	0.05 ± 0.05	0.11 ± 0.12	0.06 ± 0.04	0.16 ± 0.22^a^	0.18	0.86

HDL-c, High-density lipoprotein cholesterol; LDL-c, Low-density lipoprotein cholesterol; VLDL-c, very low-density lipoprotein cholesterol; HOMA-IR, homeostasis model assessment insulin resistance; QUICKI, quantitative insulin-sensitivity check index. adipo/lep: adiponectin/leptin ratio;

Data are presented as mean (SD);

a statistic difference between baseline and after therapy of obese adolescents (p < 0.05);

b statistic difference between baseline of adolescents (p < 0.05);

c *statistic difference between the groups in the end of therapy*.

### Effects of Therapy on Diet Patterns

The adolescents had significant decrease in energy intake after therapy, as well as reductions in dietary carbohydrate, lipids, and sodium intake. The glycemic load of the diet decreased (Table 3).

**Table 3:** Energy intake, macronutrients, micronutrients, fiber dietary, and DII before and after therapy.

**variables**	**All (*****n*** **=** **45)**		**Increased E-DII (*****n*** **=** **23)**		**Decreased E-DII (*****n*** **=** **22)**		***Effect size***	***Observed power***
	**Baseline**	**After therapy**	**Baseline**	**After therapy**	**Baseline**	**After therapy**		
Energy Intake (kcal)	1965.49 ± 830.70	1491.40 ± 623.8^a^	1894.09 ± 901.66	1532.70 ± 639.21	2040.14 ± 763.37	1448.23 ± 619.22^a^	0.21	0.92
Carbohydrate (g)	251.92 ± 106.14	181.06 ± 67.14^a^	245.10 ± 120.69	180.88 ± 48.26^a^	259.39 ± 89.94	181.25 ± 83.69^a^	0.24	0.95
Protein (g)	97.11 ± 51.29	81.24 ± 57.8	94.36 ± 49.90	90.36 ± 76.14	99.99 ± 53.72	71.70 ± 27.22	0.04	0.31
Fat (g)	25.95 ± 45.70	14.22 ± 10.17^a^	60.37 ± 32.78	50.46 ± 44.21	70.14 ± 31.29	49.28 ± 33.93^a^	0.14	0.76
Saturated Fat (g)	30.12 ± 50.24	12.92 ± 8.16	16.87 ± 11.87	15.55 ± 10.01	35.46 ± 63.59	12.83 ± 10.40^a^	0.06	0.39
Polyunsaturated fat (g)	8.64 ± 6.08	6.09 ± 4.87^a^	8.70 ± 5.86	6.31 ± 5.87	8.58 ± 6.43	5.86 ± 3.69	0.09	0.53
Fiber (g)	19.68 ± 13.4	16.05 ± 7.99	23.03 ± 13.90	15.16 ± 8.00^a^	16.18 ± 12.18	16.99 ± 8.07	0.04	0.30
Dietary Cholesterol (mg)	287.18 ± 184.41	236.5 ± 185.32	270.87 ± 181.17	263.88 ± 209.76	304.23 ± 190.45	207.87 ± 155.51	0.05	0.32
Sodium (mg)	4172.73 ± 3491.2	2634.38 ± 2264.83^a^	4389.47 ± 3824.32	3205.96 ± 2690.59	4150.68 ± 3191.44	2036.83 ± 1559.32^a^	0.22	0.93
E-DII	1.16 ± 1.52	0.83 ± 1.49	0.24 ± 1.19	1.57 ± 1.31^a^	2.21 ± 1.20	0.06 ± 1.27^a,c^	0.11	0.66
Glycemic Load	82.58 ± 51.36	59.82 ± 35.72^a^	84.13 ± 56.98	58.52 ± 29.76^a^	80.95 ± 46.06	61.18 ± 41.74	0.13	0.70
Vitamin C (mg)	102.10 ± 106.84	112.68 ± 177.30	135.91 ± 112.90	54.59 ± 47.03^a^	66.75 ± 89.45	173.42 ± 236.61^a,c^	0.04	0.07
Vitamin E (mg)	10.25 ± 7.74	6.91 ± 6.72^a^	10.98 ± 7.83	5.84 ± 5.55^a^	9.49 ± 7.76	8.03 ± 7.73	0.10	0.57
Folate (mcg)	148.64 ± 92.25	108.66 ± 68.03^a^	171.58 ± 93.19	84.57 ± 42.83^a^	124.65 ± 133.85	133.85 ± 80.47^c^	0.12	0.67

E-DII, Energy- adjusted Dietary Inflammatory Index; ^a^-P < 0.05;

a statistic difference between baseline and after therapy of obese adolescents (p < 0.05);

b statistic difference between baseline of adolescents (p < 0.05^c^) statistic difference between adolescents in the end of therapy;

c *statistic difference between the groups in the end of therapy*.

### Comparison the Effects of Therapy Between the Groups

According to the deltas analysis it was observed that body fat mass and QUICKI were statistically different between adolescents who increased DII and decreased DII after therapy. No differences were observed in the deltas of adipokines (Table 4).

**Table 4:** Delta values of the parameters analyzed.

	**All (*n* = 45)**	**E-DII increased (*n* = 23)**	**E-DII decreased (*n* = 22)**
Body weight (kg)	−5.53 ± 4.72	−4.72 ± 4.46	−6.38 ± 4.93
BMI (kg/m^2^)	−2.15 ± 1.68	−1.85 ± 1.75	−2.46 ± 1.59
Waist Circumference (cm)	−4.64 ± 5.56	−4.02 ± 3.00	−5.28 ± 7.39
Abdominal circumference (cm)	−7.75 ± 15.05	−5.22 ± 5.05	−10.40 ± 20.82
Neck circumference (cm)	−1.39 ± 1.40	−1.17 ± 0.95	−1.63 ± 1.76
Hip circumference (cm)	−4.02 ± 4.84	−4.14 ± 3.49	−3.90 ± 6.02
Body fat mass (%)	−1.51 ± 2.94	−0.65 ± 3.08	−2.41 ± 2.54^d^
Body fat mass (kg)	−3.96 ± 4.60	−2.80 ± 4.57	−5.16 ± 4.43
Body fat mass (%)	−22.75 ± 58.67	−25.60 ± 65.80	−19.77 ± 51.55^d^
Fat free mass (kg)	−1.54 ± 2.77	−1.82 ± 3.02	−1.24 ± 2.53
BMR (kcal)	−50.02 ± 85.18	−55.78 ± 92.73	−44.00 ± 78.22
Glycemia (mg/dL)	−1.80 ± 10.41	−0.22 ± 10.79	−3.45 ± 9.98
Total Cholesterol (mg/dL)	−3.56 ± 39.15	4.43 ± 40.45	−11.91 ± 36.79
HDL-C (mg/dL)	3.29 ± 6.03	3.96 ± 6.28	2.59 ± 5.81
Non-HDL (mg/dL)	−10.22 ± 39.29	−6.26 ± 44.17	−14.36 ± 34.00
LDL-c (mg/dL)	−5.71 ± 32.68	−0.39 ± 32.42	−11.27 ± 32.76
VLDL-c (mg/dL)	−2.07 ± 10.48	−1.09 ± 11.85	−3.09 ± 8.99
TG (mg/dL)	−10.29 ± 51.74	−5.91 ± 58.48	−14.86 ± 44.53
Insulin (U/dL)	−2.19 ± 11.69	−0.20 ± 15.02	−4.27 ± 6.40
HOMA-IR	−0.53 ± 2.88	−0.04 ± 3.67	4.10 ± 1.58
Adiponectin (μg/mL)	0.97 ± 3.31	1.39 ± 3.95	0.52 ± 2.48
Leptin (ng/mL)	−13.94 ± 25.56	−13.29 ± 30.35	−14.63 ± 20.06
adipo/lep	0.07 ± 0.16	0.05 ± 0.10	0.10 ± 0.21
Energy Intake (kcal)	−474.09 ± 915.54	−361.39 ± 981.69	−591.91 ± 847.47
Carbohydrate (g)	−65.26 ± 123.89	−64.22 ± 126.35	−66.34 ± 124.22
Protein (g)	−15.88 ± 72.25	−4.00 ± 89.68	−28.29 ± 46.87
Fat (g)	−15.26 ± 37.55	−9.90 ± 37.52	−20.85 ± 37.62
Saturated Fat (g)	−11.74 ± 46.86	−1.32 ± 15.11	−22.63 ± 64.17
Polyunsaturated fat (g)	−2.55 ± 8.13	−2.39 ± 8.90	−2.72 ± 7.44
Fiber (g)	−3.63 ± 16.27	−7.86 ± 16.07	0.81 ± 15.62
Dietary Cholesterol (mg)	−50.68 ± 225.22	−6.99 ± 240.15	−96.36 ± 203.94
Sodium (mg)	−1638.35 ± 3131.78	−1183.51 ± 3312.24	−2113.85 ± 2931.45
E-DII	0.41 ± 1.96	1.33 ± 0.89	−2.07 ± 1.23^d^
Glycemic Load	−22.76 ± 58.67	−25.60 ± 65.80	−19.77 ± 51.55
Vitamin C (mg)	10.58 ± 207.68	−81.32 ± 95.17	106.67 ± 248.91^d^
Vitamin E (mg)	−3.34 ± 10.12	−5.14 ± 8.84	−1.46 ± 11.20
Folate (mcg)	−39.98 ± 115.29	−87.01 ± 91.65	133.85 ± 80.47^d^

BMI (Body Mass Index), BMR (Basal Metabolic Rate); HDL-c, High-density lipoprotein cholesterol; LDL-c, Low-density lipoprotein cholesterol; VLDL-c, very low-density lipoprotein cholesterol; HOMA-IR, homeostasis model assessment insulin resistance; QUICKI, quantitative insulin-sensitivity check index; adipo/lep, adiponectin/leptin ratio;

d *Difference statistic between delta (Δ) of DII increased and DII decreased*.

For the components of dietary intake and E-DII, the deltas of Vitamin C, E, folate, and E-DII are statically different between adolescents that increased and decreased E-DII after the therapy (Table 4).

### Correlations Between Variables

**Figure 2:**
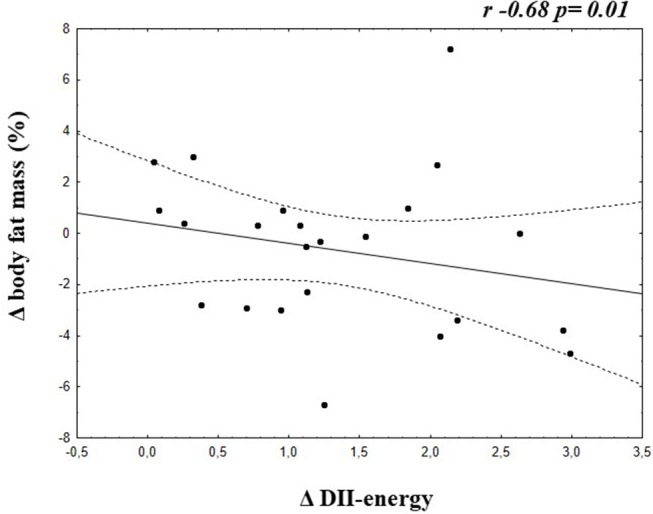
Correlation between Δ E-DII and Δ Body fat mass of adolescents who increased the energy adjusted DII.

A negative correlation was found between Δ E-DII and Δ Body fat mass (%) (−0.68; *p* = 0.01) in the group of adolescents who increased the E-DII (Figure 2).

## Discussion

The present study indicates that long-term interdisciplinary therapy coupled with web-based health education can contribute to reducing obesity and associated inflammatory processes. Indeed, the E-DII can be used to ameliorate health conditions in adolescents with obesity. In fact, we consider E-DII relevant from a clinical point of view to improve biochemical parameters and inflammation parameters (leptin and adiponectin), as well as to help to attenuate cardiovascular risks. The present investigation indicates that adolescents who observed the dietary advice tended to have lower E-DII scores, and improvements in anthropometric and inflammatory parameters compared to their counterparts who continue to eat pro-inflammatory diet.

Previous studies showed that dietary pattern and obesity could induce inflammation and increase the risk for type 2 diabetes, cardiovascular disease, and metabolic syndrome (52–54). In the present investigation, we analyzed the possible relations between the E-DII score and cardiometabolic parameters in obese adolescents.

It was found an improvement in QUICKI after therapy occurred only in adolescents who decreased the E-DII, when the sample was analyzed according the E-DII response. QUICKI is a reliable index of insulin sensitivity (44). This same group decreased glycemia, VLDL-c, triglyceride, and HOMA-IR. As previously demonstrated, insulin resistance is related to inflammatory processes, increasing risk of cardiovascular disease in obese adolescents (36, 55). It is important to know that these variables have been related to cardiovascular risks and metabolic syndrome, reinforcing the benefits of interdisciplinary therapy for the management of obesity and its related comorbidities in adolescents. Together, these results should be considered in the clinical practices.

Moreover, the therapy was effective at improving HDL-c concentrations. High level of HDL-c may contribute to cardiometabolic protection, while low HDL-c correlates with metabolic syndrome in obese adolescent (56, 57).

Additionally, the present study showed negative correlation between Δ E-DII and Δ body fat mass (%) in the group who increased the inflammatory diet profile, contributing to our understood to the importance of this E-DII in the clinical practice, as adjuvant in follow the patients to improve their health conditions.

Importantly our results show a reduction in total caloric intake as well as lower sodium, carbohydrate, and lipids intake by the end of the intervention of all adolescents. High calorie diet rich in lipids and micronutrients such as sodium can promote an increase in inflammatory indices of diet. It is well known that caloric intake higher than energy expenditure leads to body weight gain and fat deposition and that sodium intake is associated with metabolic disorders (58, 59).

Interesting, leptin is involved in the inflammatory process, and the state of hyperleptinemia may limit the adiponectin increase (9). Previous studies showed that adolescents undergoing long-term multidisciplinary therapy had significant reduction in leptin and increase in adiponectin concentration. These results are in line with our findings suggesting an attenuated inflammatory process after the proposed therapy. Although, the interdisciplinary intervention had been able to reduce hyperleptinemia state, children and adolescents should maintain healthy lifestyle to reach normal levels of this hormone (35, 60–63).

Hyperleptinemia was associated with hypoadiponectinemia, contributing to increased related inflammatory process obese adolescents (11, 35, 61, 64). Previous studies of this group showed that changes in body mass and BMI are positively correlated with decreased leptin concentration. It is important to note that a significant decrease in leptin concentration is observed when the adolescents reduced their body mass by at least 7% (55). Confirming our hypothesis, we found a decrease in the leptin concentration and inversely an increase in adiponectin/leptin ratio in all analyzed adolescents after the therapy.

Moreover, we found an increase in adiponectin/leptin ratio and a decrease in leptin on the group of decreased E-DII. This ratio could be an atherosclerotic risk maker (65) while the adiponectin/leptin ratio could be a valuable complementary element in prediction and prevention of cardiovascular diseases and metabolic syndrome (66, 67), suggesting that our therapy was effective to improve these parameters.

Other interesting results from this study include the reduction of BMI and improved body composition. This corroborates a systematic review with obese adolescents that pointed out that a multicomponent therapy, including education health were effective in improving body composition and cardiometabolic biomarkers (68, 69).

Additionally, the interdisciplinary therapy was effective in reducing waist, neck, hip, and abdominal circumferences. These circumferences are positively correlated with cardiovascular risks, obesity, and metabolic syndrome; and the improvement of these anthropometric indicators are important in controlling obesity and cardiovascular diseases (61, 67, 68, 70–74).

Faced with the above results and the improvement of fat-free mass (%), it is extremely important for health professionals to consider both the quantity and quality of the adolescent's diet as well as to encourage a change to healthy eating and active lifestyles, early in life, to prevent short-term and long-term morbidity and improve cardiometabolic parameters.

Some limitations of the present study are the lack of lean control group. Second, the Brazilian database is not complete with the 45 food parameters to calculate the E-DII, moreover these analyses were exploratory. However, our study takes future directions to investigate the inflammatory diet and the impact on metabolic and inflammatory process of obesity.

In conclusion, the long-term interdisciplinary therapy combined with web-based health education were effective in improving inflammatory markers in adolescents with obesity. In addition, these results showed that in exploratory analyses, an improvement of inflammatory and cardiometabolic parameters occurred mainly when the E-DII improved an increased inflammatory profile diet, suggesting the relevance to stimulate anti-inflammatory diet habits as an effective strategy to prevent and treat obesity and related comorbidities in adolescents with obesity.

## Ethics Statement

This study was carried out in accordance with the recommendations of Federal University to Sao Paulo ethics committee (#0448/2018) with written informed consent from all subjects. All subjects gave written informed consent in accordance with the Declaration of Helsinki. The protocol was approved by the CEP UNIFESP.

## Author Contributions

YF, AK, SV, AD, and LT designed the study. YF, AK, RC, LO, VB, and AD performed the experiments. YF, AK, RC, DM, LO, VB, AD, DT, NS, and JH analyzed the results and wrote the manuscript. LO, VB, NS, and JH contributed with reagents and analysis tools. All authors reviewed the manuscript.

### Conflict of Interest Statement

JH owns controlling interest in Connecting Health Innovations LLC (CHI), a company that has licensed the right to his invention of the dietary inflammatory index (DII) from the University of South Carolina in order to develop computer and smart phone applications for patient counseling and dietary intervention in clinical settings. NS is an employee of CHI. The remaining authors declare that the research was conducted in the absence of any commercial or financial relationships that could be construed as a potential conflict of interest.
